# Evaluation of the In Vitro Behavior of Electrochemically Deposited Plate-like Crystal Hydroxyapatite Coatings

**DOI:** 10.3390/biomimetics9110704

**Published:** 2024-11-17

**Authors:** Cosmin M. Cotrut, Alexandru Blidisel, Diana M. Vranceanu, Alina Vladescu (Dragomir), Elena Ungureanu, Iulian Pana, Mihaela Dinu, Catalin Vitelaru, Anca C. Parau, Vasile Pruna, Mihai S. Magurean, Irina Titorencu

**Affiliations:** 1 Faculty of Materials Science and Engineering, National University of Science and Technology Politehnica Bucharest, 313 Independentei Street, 060042 Bucharest, Romania; cosmin.cotrut@upb.ro (C.M.C.); elena.ungureanu1102@upb.ro (E.U.); 2 Hepato-Bilio-Pancreatic Surgery Center, University Clinic Surgical Semiology and Thoracic Surgery, “Victor Babes” University of Medicine and Pharmacy, Sq. Eftimie Murgu No. 2, 300041 Timisoara, Romania; 3 Department for Advanced Surface Processing and Analysis by Vacuum Technologies, National Institute of Research and Development for Optoelectronics—INOE 2000, 077125 Magurele, Romania; 4 Romanian Academy Institute of Cellular Biology and Pathology “Nicolae Simionescu”, 8 B.P. Hasdeu, 050568 Bucharest, Romania; 5 Colentina Clinical Hospital, 020125 Bucharest, Romania

**Keywords:** biomimetic, surface modification, hydroxyapatite coatings, electrochemical deposition, biocompatibility

## Abstract

The purpose of coatings is to protect or enhance the functionality of the substrate material, irrespective of the field in which the material was designed. The use of coatings in medicine is rapidly expanding with the objective of enhancing the osseointegration ability of metallic materials such as titanium. The aim of this study was to obtain biomimetic hydroxyapatite (HAp)-based coatings on titanium by using the pulsed galvanostatic method. The morphology of the HAp-based coatings revealed the presence of very thin and wide plate-like crystals, grown perpendicular to the Ti substrate, while the chemical composition highlighted a Ca/P ratio of 1.66, which is close to that of stoichiometric HAp (1.67). The main phases and chemical bonds identified confirmed the presence of the HAp phase in the developed coatings. A roughness of 228 nm and a contact angle of approx. 17° were obtained for the HAp coatings, highlighting a hydrophilic character. In terms of biomineralization and electrochemical behavior, it was shown that the HAp coatings have significantly enhanced the titanium properties. Finally, the in vitro cell tests carried out with human mesenchymal stem cells showed that the Ti samples coated with HAp have increased cell viability, extracellular matrix, and Ca intracellular deposition when compared with the uncoated Ti, indicating the beneficial effect.

## 1. Introduction

Enhancing the biointegration of orthopedic and dental implants in the surrounding tissues is crucial and a primary objective in medicine to ensure the successful functioning of implanted materials and devices within the human body [1]. Most metal implants that are currently approved and widely used in medicine are made of pure titanium or its alloys, such as Ti6Al4V, because they are preferred for load-bearing applications due to their superior mechanical properties, namely high strength, proper fracture toughness, and good corrosion resistance [2,3]. 

However, metallic implants are susceptible to corrosion, which favors the release of small metallic particles into the human body that can cause chronic inflammation [4,5]. Furthermore, it has been shown that metallic implants are insufficiently biologically active regardless of their proper mechanical properties [6]. To overcome this inconvenience, the metallic surface can be functionalized with a bioactive material. In this manner, the surface characteristics of a metallic material can be modulated to achieve the desired features [7,8,9]. Hydroxyapatite (HAp) is the most known bioactive ceramic that can be used as a coating due to its similarity to the mineral phase of bone, which can favor the occurrence of the biochemical bond at the implant–bone interface [10,11,12].

So far, several innovative biomaterials, surface modification methods, and coatings have been developed with the aim of enhancing the biological response of metallic implants [13,14,15]. One way to modulate the interaction at the metallic implant–tissue interface is to use a coating made of a biomimetic material such as hydroxyapatite to enhance the overall characteristics of Ti, especially the biological ones. In this regard and considering the applicability of bioceramic materials in medicine, Sigumoto et al. [16] stated that materials that promote bone formation and are compatible with bone replacement and therapy should resemble bone, present a low crystallinity, and have a nanocrystalline structure. 

Hydroxyapatite-based coatings can be prepared by various methods, which can be divided into solid-state reactions and wet chemical methods, each having its advantages and disadvantages and being well presented and discussed in the literature [17,18]. In terms of morphology, the literature reports that hydroxyapatite can present diverse types of morphologies [19]. Considering the main fabrication parameters involved in the wet chemical methods, HAp exhibits a hexagonal symmetric structure from which needle-like and plate-like structures can be developed by using the Ca/P = 1.67 stoichiometric ratio. 

Among the most used methods to obtain hydroxyapatite with plate-like morphology are the sol–gel route [20], the hydrothermal method [21], wet chemical precipitation [22], the biomimetic deposition method [23], and electrochemical deposition [24,25,26].

The electrolytic deposition method, also known as electrodeposition, has gained significant attention for depositing HAp coatings due to its ability to precisely control the coating’s thickness, but also its composition and morphology [24,25,26,27]. In the case of plate-like hydroxyapatite, this approach offers novel capabilities that distinguish it from conventional HAp deposition techniques, especially in terms of controlling the crystal orientation, morphology, and overall bioactive properties. Table 1 summarizes the main electrochemical parameters involved in the deposition process of HAp with plate-like morphology.

Considering the data presented in Table 1 and the material science tetrahedron that emphasizes the interplay of four distinct aspects, namely ***processing–structure–properties–performance***, it becomes clear that even the smallest change in the experimental design can lead to new findings and/or enhanced properties. Thus, material performance can be modified by changing the material structure through engineering approaches to deliver the desired performance. 

Electrochemical deposition allows fine-tuning of the deposition parameters, such as current density, voltage, electrolyte concentration, and temperature, which directly influence the morphology of the deposited HAp. The novelty of depositing plate-like hydroxyapatite using this method lies in its ability to produce HAp crystals with a highly oriented and elongated structure. These plate-like structures resemble the natural apatite crystals found in human bone, making them ideal for mimicking bone-like properties. 

Currently, there is a high interest in mimicking natural bone, which consists of hydroxyapatite crystals with a plate-like aspect with dimensions of 40–60 nm length and 20–30 nm width [42,43]. Despite disagreements regarding the crystal morphology that date back to the 1950s [44,45], it is widely acknowledged that the bone apatite nanocrystal exhibits a plate-like shape that is elongated parallel to the *c*-axis of the hexagonal apatitic structure [46] instead of a needle-like shape. The physico-chemical properties of a material can be modified by carefully designing the experimental set-up, which in the case of electrochemical deposition are the electrolyte’s concentration and or pH value, the applied potential or current density, and so on [47,48,49]. 

The plate-like morphology of HAp obtained via electrodeposition enhances osseointegration, as the surface structure more closely mimics the nanostructure of natural bone. The flat, extended surfaces of plate-like HAp provide more surface area for cell attachment, improving osteoblast adhesion and proliferation. Additionally, this structure can create interconnected porosity on the coating surface, further enhancing bone tissue ingrowth [25,37].

Unlike methods such as plasma spraying, which often require elevated temperatures that can degrade the bioactivity of HAp, electrodeposition occurs at lower temperatures (<95 °C) [37]. This allows the deposition of plate-like HAp while maintaining its biological properties, ensuring that the coating remains bioactive and capable of promoting bone cell activity post-deposition. The low-temperature process also enables the coating of heat-sensitive substrates [33,36], such as polymers or hybrid materials, further expanding the application of plate-like HAp coatings [50]. Moreover, a large variety of elements can be added within the HAp structure to offer antibacterial efficiency (Zn, Ag, Cu) to support bone growth in osteoporotic bone (Sr) or to enhance the biodegradability (Mg) of the coatings [11,51,52,53,54]. One other advantage of this technique is the large variety of morphologies, such as plate-like, needle-like, rods, and flowers, that can be obtained by modifying the driving (applied potential or current, deposition time, electrolyte pH and/or temperature or concentration) or the assisting (substrate modification and pulsed/cyclic deposition) parameters used in the electrochemical deposition process. 

Novel research has shown that plate-like HAp structures, when electrodeposited on metal substrates (e.g., titanium or stainless steel), offer enhanced corrosion protection in physiological environments [24,40]. The aligned plate-like structure acts as a barrier to corrosive ions, while the uniform and tightly bound coating improves the mechanical stability of the implant. This makes plate-like HAp coatings especially attractive for long-term implants that require both mechanical durability and resistance to degradation in the body.

The deposition of plate-like hydroxyapatite using the electrolytic deposition method represents a novel and promising approach in the field of biomaterials. By leveraging the ability to control the crystal structure, morphology, and thickness of HAp coatings, features such as osseointegration, mechanical properties, and functional versatility (Table 1) can be improved. The combination of low-temperature processing and applicability to complex implant geometries makes this method a significant advancement in the development of next-generation biomedical implants.

In a study performed by Marashi-Najafi et al. [25], it was shown that in comparison with the needle-like morphology, the HAp-based coatings with plate-like crystals showed the highest cell density and cell proliferation after 5 days of seeding with fibroblast and a higher biomineralization ability in simulated body fluid (SBF), even though both types of coatings presented values of the contact angle of 21.9° for the plate-like crystals and of 14.1° for the needle-like crystals, indicating a hydrophilic character. Furthermore, in terms of chemical stability, the same study [25] showed that plate-like morphology has a lower dissolution rate in physiological saline solution. Another study performed in vitro with mouse fibroblast cells has also shown that the hydroxyapatite-based coatings with plate-like morphology present a higher number of attached cells and cell density, in comparison with the HAp coatings with needle-like morphology [55].

Hydroxyapatite-based coatings represent a critical technology in the field of biomedical implants, offering biocompatibility, bioactivity, and mechanical performance. Advances in deposition techniques and functionalization strategies have broadened the scope of HAp coatings beyond traditional applications. As research continues to address the challenges of mechanical stability, in vivo durability, and multifunctionality, hydroxyapatite coatings will likely play an even more prominent role in the future of implantable medical devices.

The goal of this study was to electrochemically deposit hydroxyapatite (HAp)-based coatings with a plate-like morphology, similar to the natural HAp found in the hard bone tissue, and to illustrate that the proposed technique is highly reproducible. The obtained coatings were physico-chemical characterized in depth and the in vitro behavior in terms of bioactivity, electrochemical behavior, and cell interaction was determined. Although intensive studies have covered plane HAp properties and application areas, to the best of our knowledge, the proposed experimental design and the impact of this morphology— namely plate-like, which is thought to be biomimetic—on the coating’s properties have not yet been explored.

## 2. Materials and Methods

### 2.1. Electrochemical Deposition of Hydroxyapatite 

Titanium was used as a substrate material to be coated with ceramic hydroxyapatite using the electrochemical deposition (ED) technique. The substrates were cut as discs with a height of 2 mm from a titanium bar (Bibus Metals Ag, Essen, Germany) with a diameter of 20 mm. The resulting discs were prepared on SiC paper of different grits (200 P–800 P), cleaned for 30 min in acetone in an ultrasonic bath (Bandelin, Berlin, Germany), and then washed with ultra-pure water before the electrochemical deposition. 

The electrochemical deposition of the hydroxyapatite coatings on the titanium surface was carried out using a potentiostat/galvanostat PARSTAT MultiChannel equipped with a 2000 module (Princeton Applied Research, Ametek, Oak Ridge, TN, USA). The electrochemical cell was set up with three electrodes in which the titanium discs were used as working electrodes, a saturated calomel electrode (SCE) was the reference electrode, and a Pt plate was used as a counter electrode. The electrolyte solution used to obtain the ceramic coatings was prepared by subsequently dissolving 2.5 mM of Ca(NO_3_)_2_·4H_2_O and 1.5 mM of NH_4_H_2_PO_4_ in ultra-pure water ASTM I. All chemicals are of high purity and were purchased from Sigma Aldrich (Munich, Germany). The electrolyte pH was set to 6 by the addition of 40 μL of 1M NaOH. The deposition was carried out at a constant temperature of 60 °C (±0.5 °C) under continuous stirring of the electrolyte with a speed of 100 rpm with a basic magnetic hotplate stirrer (KA RCT Basic Safety Control Hotplate/Stirrer and ETS-D6 Temp, IKA, Staufen, Germany). 

The pulsed galvanostatic technique was used for the electrochemical deposition of the ceramic coatings and the parameters involved in the process were controlled with VersaStudio software (version 2.62.2). A total of 300 cycles were used for each deposition, and one cycle consisted of a current density of −3 mA/cm^2^ applied for 1 s to activate the ions found in the electrolyte, followed by a 5 s period in which the current density was zero. 

### 2.2. Coating Characterization and Testing 

#### 2.2.1. Physico-Chemical Investigations 

A scanning electron microscope equipped with an X-ray energy-dispersive spectrometer (SEM-EDS, Phenom ProX, Phenom World, Eindhoven, The Netherlands) was used to analyze the morphological aspects of the developed coatings.

X-ray diffraction (XRD) data were acquired using a SmartLab X-ray diffractometer (Rigaku, Tokyo, Japan) equipped with a 9 kW Cu rotating anti-cathode, a vertical goniometer of 300 mm radius with sample horizontal mount, and a HyPix 3000 two-dimensional semiconductor detector. Grazing incidence measurements were performed in the 20–80° range with a resolution of 0.3°, and Rigaku’s PDXL software package (version 2) was used to analyze the diffractograms. 

To highlight the chemical bonds, a spectrophotometer FT-IR, Jasco 6300 (Jasco, Tokyo, Japan), equipped with a universal ATR sampling accessory Pike MIRacle (Pike Technologies, Madison, WI, USA) was used. The IR spectra were acquired at a resolution of 4 cm^−1^ over the wavelength range of 500–4000 cm^−1^.

The sessile drop method was used to determine the contact angle (CA) by using a KSV-Instruments Attention TL101 goniometer (Biolin Scientific, Stockholm, Sweden). The measurements were carried out at ambient conditions (temperature of 25 ± 0.5 °C, 40% relative humidity) and a medium simulated body fluid (SBF, chemical composition: 7.996 g/L NaCl, 0.350 g/L NaHCO_3_, 0.224 g/L KCl, 0.228 g/L K_2_HPO_4_·3H_2_O, 0.305 g/L MgCl_2_·6H_2_O, 40 mL 1 M-HCl, 0.278 g/L CaCl_2_, 0.071 g/L Na_2_SO_4_, and 6.057 g/L Tris-NH_2_C(CH_2_OH)_3_) was used. A 5 μL droplet of SBF was applied with a Hamilton micro-syringe on the material surface, and a charge-coupled device (CCD) camera recorded the contour. The contact angle was measured in triplicate and the data are presented as the means ± standard deviation (SD). 

The DEKTAK 150 stylus profilometer (Veeco Instruments, Plainview, NY, USA) was used to measure the surface roughness of the developed coatings over a range of 3000 μm. Ten measurements were carried out on each sample, and the tests were carried out on three different samples. Based on the profiles obtained, the main parameters, namely Ra—the average surface roughness, Rq—the root mean square roughness, and Rsk—the factorial asymmetry of the evaluated surface, were obtained and the results are presented as the means ± standard deviation (SD). 

#### 2.2.2. Electrochemical Behavior in SBF 

The ceramic coatings’ electrochemical behavior in the SBF medium was assessed using the linear polarization technique. The experiments were conducted using a PARSTAT 4000 potentiostat (Princeton Applied Research, Ametek, Oak Ridge, TN, USA) equipped with a low-current interface module (Princeton Applied Research, Ametek, Oak Ridge, TN, USA). The investigated materials were set as working electrodes, a Pt foil was used as the counter electrode, and a saturated calomel electrode (SCE) served as the reference electrode in this three-electrode electrochemical cell. The experiments were conducted at a constant temperature of 37 °C (±0.5 °C) using a heated circulating bath (CW-05G, Jeio Tech, Yuseong-gu, Daejeon, Republic of Korea).

The open circuit potential (OCP) was monitored in SBF until the equilibrium state was reached. The tests were conducted in triplicate and all measurements were carried out at a scanning rate of 0.167 mV/s in accordance with the ASTM G5–94 standard [56]. 

#### 2.2.3. Apatite-Forming Ability 

In vitro biomineralization tests were conducted according to the ISO 23317/2014 standard and were used to assess the apatite-forming ability [57]. Briefly, each sample was individually immersed in 50 mL of SBF and each day the solution was renewed to maintain the ionic composition. The tests were conducted at 37 °C (±0.5 °C), which was held constant with an incubator (Memmert, IF 55, Schwabach, Germany) for 21 days. At predefined time intervals (1, 2, and 3 weeks), one sample was removed, rinsed with ultra-pure water, and dried in a desiccator until required for further investigation. Using an analytical balance (Kern, ALT 100-5AM, Balingen, Germany) with an accuracy of 0.01 mg, the mass evolution of the samples was assessed. Energy-dispersive spectrometry (EDS, Bruker, Billerica, MA, USA) was used to assess the chemical composition of the material, while a scanning electron microscope (SEM, Hitachi TM3030Plus, Tokyo, Japan) was used to examine the surface morphology of the material. 

#### 2.2.4. Cell Culture of BMSCs on cp-Ti Substrate HAp Coating

Having the approval of the Institutional Ethical Committee (180/27 September 2018), human bone marrow mesenchymal stem cells (BMSCs) were isolated after obtaining informed consent. This procedure adhered to the World Medical Association’s Declaration of Helsinki (Ethical principles for medical research involving human subjects, November 2013) [58]. This study included patients who underwent surgery for osteoarthritis-related complications. The isolation of human bone marrow-derived MSC was performed using a modified protocol that our group had previously established and published [59].

For all in vitro experiments, the cells were seeded at a density of 9 × 10^3^ cells/cm^2^, and the tests were performed 5 days after cell seeding on the cp-Ti and HAp coating samples. To achieve an equal cell seeding density on the sample surface, they were measured using an electronic caliper, and the necessary volume of suspension as well as its cellular density was determined according to the previously published method [60].

The samples were first sterilized by maintaining them for 24 h in 70% vol/vol ethyl alcohol and then rinsed 3 times with sterile endotoxin-free water. Then, the cells were incubated for 2 h in DMEM with a low glucose culture medium (Sigma Aldrich St. Louis, MO, USA) with 10% fetal bovine serum. 

#### 2.2.5. Evaluation of Cell Viability and Proliferation 

BMSC viability was determined by the MTT (3-(4.5-dimethylthiazol-2-yl)-2.5 diphenyl tetrazolium bromide) assay (Sigma Aldrich, St. Louis, MO, USA). Cells cultured on the surfaces of the samples were rinsed with warm phosphate-buffered saline (PBS) and incubated for 2.5 h with 0.5 mg/mL MTT solution. The assay is based on reducing the tetrazolium salt by the metabolically active cells to a blue formazan, which is then solubilized with 0.1 N HCl in anhydrous isopropanol. The absorbance at 570 nm (with reference at 630 nm) was measured using a Tecan spectrophotometer.

Cell viability was also highlighted by fluorescence microscopy using the Life Death kit (Thermo Fisher Scientific, Waltham, MA, USAn all of these cases, the cells were seeded at a density of 9 × 10^3^ cells/cm^2^, and the tests were performed 5 days after seeding on the cp-Ti and HAp coating samples.

The proliferation was determined by measuring the amount of deoxyribonucleic acid (DNA) released by the cells after breaking the cell membranes following large temperature variations. Briefly, the cells were rinsed with warm PBS and then subjected to 4–5 large temperature variations using liquid nitrogen. After the last freeze–thaw cycle, a Hoechst 33258 (Sigma Aldrich, St. Louis, MO, USA) fluorochrome solution with a concentration of 10 μg/mL in TNE buffer consisting of 10 mM TRIS, 1 mM EDTA, and 2M NaCl (pH 7.4) was added to the cells and incubated for an hour at 37 °C. After incubation, the emitted fluorescence was measured. The excitation of the Hoechst 33258 fluorochrome was conducted at λ = 350 nm and the fluorescence emission was recorded at λ = 460 nm. Using the standard curve made with salmon DNA (ranging between 25 and 5000 ng DNA) in TNE buffer and the fluorescence measurements of the samples, the amount of released DNA from the cells was determined. 

#### 2.2.6. Immunofluorescence Staining

BMSCs were cultured for 5 days on the cp-Ti and HAp coating samples. Following cell permeabilization using 0.1% Triton X, the assessment of vimentin and type I collagen expression was performed. The cells underwent a PBS rinse before being treated with specific antibodies: anti-type I collagen (Thermo Fisher Scientific) and anti-vimentin (Sigma-Aldrich, St. Louis, MO, USA). As a secondary antibody, anti-mouse Alexa 488 antibody (Thermo Fisher Scientific St. Louis, MO, USA) was used. The nuclei were stained with DAPI (Sigma Alrich) and the images were observed under a fluorescence microscope (Zeiss Observer D1, Oberkochen, Germany).

#### 2.2.7. Actine Cytoskeleton Staining

FITC (Fluorescein 5(6)-isothiocyanate)-Phalloidin was used to visualize actin staining in BMSCs grown for one and five days on the Ti and HAp coating samples. The cells were washed with PBS twice and then incubated for 15 min with 0.1% Triton-X 100 (Sigma Alrich St. Louis, MO, USA) and 4% PFA solution. Then, the cells were incubated with 10 µg/mL FITC-labeled Phalloidin (Sigma Alrich St. Louis, MO, USA) for 1 h. 

#### 2.2.8. Alkaline Phosphatase (ALP) Activity Assay

Alkaline phosphatase activity was determined by using p-nitrophenyl phosphate as an enzyme substrate and measuring the amount of transformed substrate. Briefly, the cultured cells were gently washed with warm PBS and the substrate consisting of p-nitrophenyl phosphate solution (9.88 mM) dissolved in an alkaline buffer was added over the cells and placed into an incubator for 60 min. A standard curve was made using dinitrophenol (ranging between 20 and 200 μM). After incubation, the supernatant was collected and the absorbance of both samples and the standard curve was measured at λ = 405 nm. 

#### 2.2.9. Scanning Electron Microscopy (SEM)

The samples were processed after 5 days to examine cell morphology. Briefly, the cells were rinsed with warm PBS and fixed with warm 4% formaldehyde in Cacodylate buffer at room temperature for 15 min. After a PBS wash, the samples were subjected to dehydration and prepared for scanning electron microscopy (SEM).

### 2.3. Statistical Analysis

The data are presented as the means ± standard deviation (SD). All experiments were performed in triplicate (*n* = 3) and the results are shown from one representative experiment. Statistical analysis was performed using Student’s paired t-test, with a two-tailed distribution. The high significance level was set at a probability value (*p*-value) < 0.001, the significance level was set at *p*-value ≤ 0.05, and the non-significance level was set at *p*-value > 0.05. 

## 3. Results and Discussion

### 3.1. Morphology 

**Figure 1:**
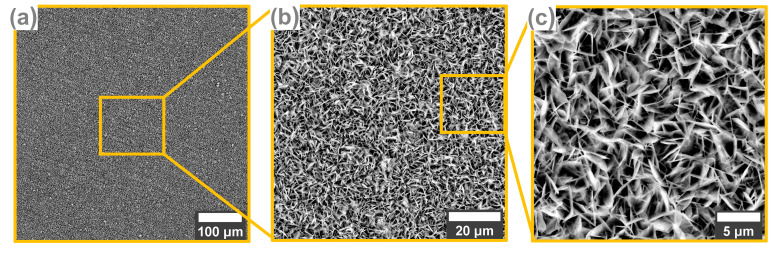
Morphology of the HAp-based coatings obtained by SEM.

Figure 1 depicts the SEM images of the hydroxyapatite coating obtained through the electrochemical galvanostatic pulse technique. The image obtained at low magnification (Figure 1a) reveals a completely covered surface with few agglomerations and no defects. At higher magnifications (Figure 1b,c), the morphology consists of small plate-like crystals, which are wide and very thin. In recent years, the trend in tissue engineering has been to develop biomaterials with nanometric dimensions because they have a much larger contact surface compared to those of micrometric dimensions. This surface leads to a high surface-to-volume ratio and wetting capacity, inhibiting the development of bacteria such as *E. coli*, *S. aureus,* and *P. aureginosa* at the same time [61,62]. Moreover, another aspect revealed by the SEM images is the preferential orientation of the plate-like crystal, which is perpendicular to the surface of the metallic substrate. 

According to the literature, this type of morphology is characteristic of potentiostatic/dynamic techniques [28,63], but not limited to, since the morphology of hydroxyapatite coatings depends on all parameters involved in the deposition process. Fathyunes et al. [64] obtained plate-like morphology through this technique by applying a current density of 15 mA/cm^2^, while Marashi-Najafi et al. [65] deposited HAp coatings on NiTi alloy at three different current densities (1.5 mA/cm^2^, 3 mA/cm^2^, and 5 mA/cm^2^) and the plate-like morphology was obtained when the current density was at least 3 mA/cm^2^. Thus, this morphology that mimics in terms of shape, the natural hydroxyapatite found in the hard bone tissue can also be obtained by pulse galvanostatic deposition when a current density of 3 mA/cm^2^ is applied. 

On the other hand, the nucleation process of HAp coatings depends on the deposition time as well as the electrolyte’s concentration. Mokabber et al. [66] noticed that the plate-like morphology is developed in the first five minutes of the deposition process, while a higher deposition time leads to the formation of ribbon-like crystals. Regarding the electrolyte concentration, it was observed that a low concentration of Ca^2+^ leads to a rod-like morphology, while a high one leads to the formation of ribbon-like crystals, with an intermediate step between that favors the formation of plate-like crystals [27]. Further details regarding the mechanism of the growth of this type of morphology with respect to different types of parameters are well described in the literature [48,49,51,67].

Thus, by controlling the deposition parameters, such as electrolyte concentration, current density, and deposition time, this specific morphology consisting of plate-like crystals can be obtained on Ti. 

Comparing the experimental design of this research with the ones found in the literature [30,31,68], it can be noted that HAp-based coatings can also be obtained by using a current density of −3 mA/cm^2^, a deposition temperature of 60 °C, an electrolyte with a pH value of 6, and low concentrations of Ca(NO_3_)_2_·4H_2_O (2.5 mM) and NH_4_H_2_PO_4_ (1.5 mM).

### 3.2. Chemical Composition 

**Figure 2:**
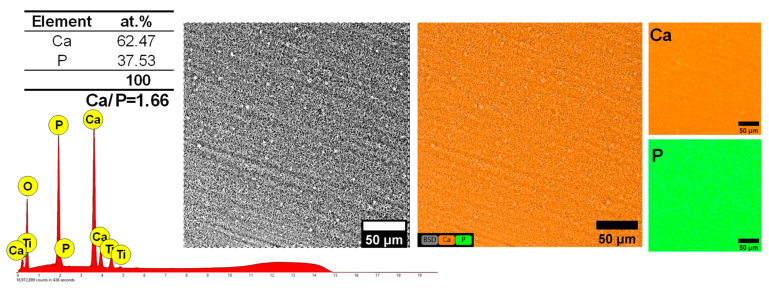
EDS analysis achieved on the HAp with plate-like morphology.

Hydroxyapatite is one of the most stable forms of calcium phosphate with a Ca/P ratio of 1.67. As the value of this ratio decreases, so does its stability in the physiological environment [69]. Figure 2 presents the EDS spectra, chemical composition, and elemental distribution of the HAp-based coatings deposited on the titanium substrate. 

Thus, from the EDS analysis presented in Figure 2, it was highlighted that both Ca and P are present in the coatings, and from the point of view of the Ca/P ratio, a value of 1.66 (±0.02) was achieved, which is very close to that of the stoichiometric HAp of 1.67. Usually, the Ca/P ratio in the case of HAp with plate-like morphology is between 1.10 and 1.60 [31,34,40,41] and in some cases reaches a value between 1.61 and 1.70 [25,31]. Thus, it can be assumed that by using the proposed electrochemical deposition set-up, a higher Ca/P ratio can be reached.

Regarding the elemental distribution, it was observed that both constitutive elements, Ca and P, were uniformly distributed on the analyzed surface. The EDS spectra also revealed the presence of Ti from the substrate along with the presence of O, which can be associated with the titanium oxide that naturally exists on the titanium surface and/or from the hydroxyapatite (chemical formula: Ca_10_(PO_4_)_6_(OH)_2_)) coating. 

### 3.3. Phase Composition 

**Figure 3:**
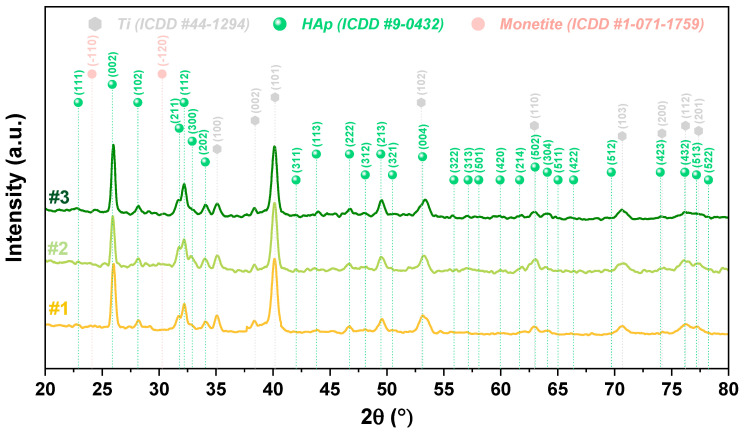
Diffractograms of 3 different HAp samples with plate-like morphology.

Figure 3 depicts the diffractograms obtained for three different samples of HAp-based coatings with plate-like crystals electrochemically deposited on Ti substrate. It is worth noting that regardless of the investigated sample, the diffraction peaks are similar, indicating that the process is a reproducible one. Also, the diffraction peaks related to the HAp structure were observed in accordance with the ICDD PDF #09-0432, indicating the formation of a stoichiometric apatite structure, having the most intense diffraction peak at an angle 2θ of ~26° (25.88°), related to the reflection plane (002). This result shows that the preferential orientation of these coatings is along the *c*-axis direction, namely perpendicular to the cp-Ti substrate, which is specific to electrochemical depositions [70]. Additionally, coatings with a preferential orientation along the *c*-axis direction can favor the bone remodeling process and offer higher stability in biological environments [71].

According to ICDD card No. 01-071-1759, two small diffraction peaks at an angle 2θ of 24.10° and 30.26° were associated with the presence of monetite, which can improve the biodegradation rate of hydroxyapatite, thus accelerating the osseointegration of implants by locally increasing the concentration of calcium and phosphate ions [72]. 

Also, the diffraction peaks corresponding to titanium were identified according to ICDD card no. 044-1294. The presence of these peaks suggests that the coatings are either thin or porous since the investigations were conducted in grazing mode. 

Next, the crystallite dimension and crystallinity were calculated. The crystallite dimension (L_(002)_) was calculated for the (002) diffraction plane, which is the most intense diffraction peak in HAp coatings obtained by electrochemical deposition, by using the Debye–Scherrer equation [70], while the crystallinity (χ_c_) was estimated by using the following equation: χ_c_ = (K_A_/β)^3^, where K_A_ is a constant equal to 0.24 for HAp and β is the FWHM of refection (002) in degrees [70]. The results revealed that the coatings registered a crystallite dimension of 21.39 nm (±0.23 nm) and a crystallinity of 25.99% (±1.14%), which is remarkably close to other results [37]. Additionally, the literature [73,74] suggests that the crystallite size along the *c*-axis in human bones is between 21 and 25 nm, which is in line with the results obtained. The crystallinity of HAp coatings could be improved through alkaline treatment before the deposition or by adding H_2_O_2_ to the electrolyte solution [75]. 

### 3.4. Chemical Bonds 

**Figure 4:**
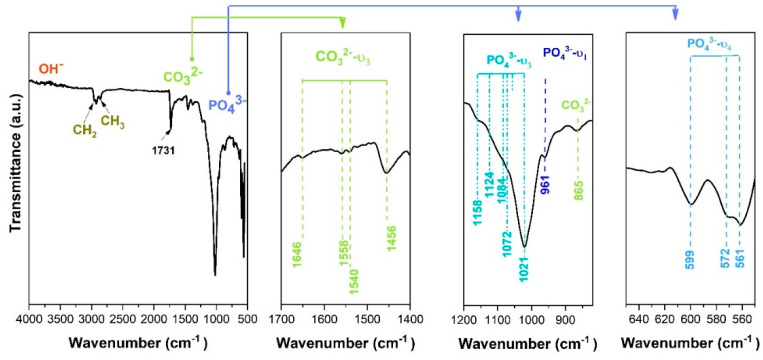
FTIR spectra of HAp coatings with plate-like morphology.

The chemical bonds obtained through Fourier-transform infrared spectrometry (FTIR) are presented in Figure 4. Initially, the coating was peeled off the substrate and the FTIR analysis was carried out on the resulting powder. 

The FTIR spectra revealed the specific bands corresponding to phosphate, carbonate, and hydroxyl groups. The vibration bands registered in the 550–604 cm^−1^ spectral range are attributed to the asymmetric stretching vibrations of the phosphate group and belong to the O-P-O bending mode, PO_4_^3−^ (ν_4_) [76,77]. The PO_4_^3−^ (ν_1_) band highlighted at 961 cm^−1^ arises from the symmetric stretching mode and the weak intensities, characteristic of apatite, might indicate a B-type substitution of hydroxyapatite [78]. The vibration mode of PO_4_^3−^ attributed to the ν_3_ vibrational mode was also identified in the spectral range of 1021–1158 cm^−1^. The vibrational modes identified include C-H deformation at 2857 cm^−1^ (CH_2_ asymmetric stretching), 2925 cm^−1^ (CH_3_ symmetric stretching), and 2955 cm^−1^ (CH_2_ symmetric stretching) due to the conductive carbon adhesive tape used to secure the peeled-off coating [79]. 

The characteristic vibration modes of the CO_3_^2−^ group were identified between 1400 and 1690 cm^−1^. Through FTIR only, the carbonate ν_2_ and ν_3_ can be highlighted. In the case of carbonated hydroxyapatite formation, carbonate ions can substitute either the hydroxyl groups OH^−^ or phosphate ions PO_4_^3−^ to give A-type or B-type carbonated HAp, respectively [80]. Conventionally, preparation of A-type HAp requires higher temperatures (800–1000 °C and a dry CO_2_ atmosphere), while the B-type HAp can be obtained by wet methods using precipitation or hydrolysis reactions at low temperatures (20–120 °C) [81].

Therefore, the vibration band registered between 1400 and 1690 cm^−1^ can be attributed to the asymmetric stretching ν_3_ mode. Moreover, the peak at ~870 cm^−1^ can prove the presence of CO_3_^2−^ stretching mode or that of the HPO_4_^−^ group [82,83]. Given that the HPO_4_^−^ functional group partially covers the one of CO_3_^2−^, it can be said that it is quite challenging to appreciate which group this band belongs to. In the literature, it is mentioned that a combination of the CO_3_^2−^ group identified at 1456 cm cm^−1^ and ~870 cm cm^−1^ proves the B-type substitution through the substitution of phosphate with carbonate in the HAp lattice [82,84]. As a rule, carbonated HAp is characterized by the division of this vibration mode into two vibration bands [78,85]. Also, the presence of a vibration band at 1730 cm^−1^ denotes a B-type substitution (the substitution of phosphate ions) [86].

The FTIR spectra showed an absorption peak corresponding to the hydroxyl group (OH^−^) starting at a wavenumber of 3450 cm^−1^, which is specific to the stretching vibration frequently associated with water adsorbed in the hydroxyapatite structure.

The presence of vibration modes typical of carbonate and their intensities indicated a carbonated HAp. However, it can be observed that the ν_3_ vibration mode is characterized by the presence of vibration bands in this range, which also demonstrates the formation of HAp. 

It is known that hydroxyapatite in human bones and teeth is not stoichiometric or pure, but it contains various elements including carbonates CO_3_^2−^ exhibiting a higher solubility compared to stoichiometric hydroxyapatite, enhancing properties such as biodegradability [87]. 

### 3.5. Roughness and Wettability 

The surface roughness has a significant influence on the osseointegration process, inducing a favorable cellular response at the protein-surface and cell-surface interface [88]. Additionally, the surface roughness influences the adhesion, proliferation, and differentiation of osteoblasts [89,90]. It has been demonstrated that osteoblasts adhere very quickly to a rough surface [17,18]. On the other hand, Orsini et al. [91] and Schwartz et al. [92] have observed that the surface roughness influences the proliferation, differentiation, and local production of osteoblasts, which, in turn, depend on the activation of some enzymes on the metal substrate, such as phosphokinase type C (PKC) and type A (PKA) and phospholipase type C (PLC) and type A_2_ (PLA_2_) [93]. Also, rougher surfaces are preferred for medical devices [94], as they ensure better adhesion of monocytes than that offered by smoother surfaces. In this regard, the literature reveals that the roughness can be classified at a larger scale into macro-roughness (100 µm–100 mm), micro-roughness (100 nm–100 µm), and nano-roughness (<100 nm) [95], or at a smaller scale into smooth (average roughness *R*_a_ < 0.5 μm), machined/minimal (0.5–1 μm), moderate (1–2 μm), and rough (>2 μm) [96,97]. Following the above, it appears that compared to a very rough or a very smooth surface, one with moderate roughness favors the growth of peri-implant bone [98]. Also, another important aspect to be considered is bacterial film formation since an optimal value of roughness will not only favor the proliferation of osteoblast cells but also the proliferation of bacteria and fungi. Biofilm formation can be minimized if the roughness is within the nanometric scale [14]. However, it is difficult to compare the results with other studies, as some studies have only performed qualitative analyses. 

**Figure 5:**
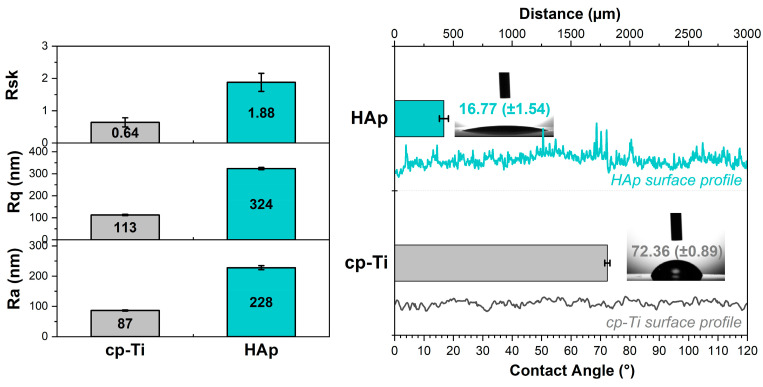
Roughness profiles and parameters and the contact angles obtained for the titanium uncoated and coated with hydroxyapatite.

According to the results obtained (Figure 5), all three samples have a similar roughness. The HAp coatings recorded a value of the Ra parameter of approx. 228 nm, which is about three times higher than that of the cp-Ti substrate, which registered a value of approx. 87 nm. Thus, it can be noted that the HAp coatings recorded a higher surface roughness due to the growth of plate-like formations. 

The skewness parameter (Rsk) describes the asymmetry of the roughness profile, which can register positive or negative values. The positive values are registered when the surface consists of more hills than valleys. Thus, the closer the value of the Rsk is to 0, the smoother the surface is. Based on Figure 5, it can be noted that both investigated surfaces presented Rsk parameter values higher than 0, indicating that both surfaces are predominant in hills. Thus, the cp-Ti surface registered an Rsk of 0.64 while the HAp coatings had a value of 1.88, showing that the coatings led to higher hills. The increment of the Rsk parameter for the HAp coatings is associated with plate-like morphologies, which led to a three-dimensional architecture. Positive values (above zero) and the increment of the skewness parameter are considered to have a beneficial impact on corrosion resistance [99]. 

The surface roughness and contact angle are some of the most important characteristics of the surface. The wetting degree of a surface can be evaluated by determining the contact angle, which is the angle at which the interface of a liquid meets the solid surface. It is widely known that a surface exhibiting a hydrophilic character (contact angle is smaller than 90°) promotes cell adhesion and proliferation and can sustain the various stages of the osseointegration process [100]. 

Nevertheless, it is difficult to predict the correct value of the contact angle that promotes the cell–implant surface interactions, as some studies revealed that a CA between 30° and 70° favors viability, spreading, and proliferation, while others have suggested that there is no predictive correlation between cell interactions and a contact angle value between 37° and 80° [101]. Therefore, creating a suitable surface state in terms of a customized surface topography and chemistry that promotes bone cell growth and permits optimal osseointegration within the bone tissue is a crucial factor for implantable materials [102]. The modified surfaces offer the rare ability to directly influence the molecular and cellular processes—such as protein adsorption, cell adhesion, and proliferation—that ultimately dictate the total biological response to an implanted material.

In the present study, the contact angle was measured using SBF as a liquid on three different samples, based on which the mean values and standard deviation were calculated. 

The obtained results showed that the uncoated Ti substrate has a registered contact angle value of 72.36° (±0.89°), which is in accordance with the literature [51], while the Ti substrate coated with HAp led to a contact angle of 16.77° (±1.54°), indicating that obtained HAp coatings are hydrophilic and have a very high wetting degree. These results are in good correlation with other studies [25,68], which have shown that the contact angle decreases due to the typical topography of electrochemical hydroxyapatite coatings. Thus, the decrease in the contact angle of the HAp coatings can also be correlated with surface roughness, which registered a much higher value of the Ra parameter than uncoated Ti.

In a study performed by Mehrvarz et al. [40], the plate-like HAp morphology presented an average roughness of ~208 nm, which is very close to the one presented above (Ra = 228 nm), and a contact angle of 31°, which is more than 50% higher than the one obtained in the current study (CA = 16.77°). Based on this, it can be hypothesized that even though both coatings present plate-like morphology, the parameters involved in the electrochemical deposition technique can lead to different properties. 

### 3.6. Electrochemical Behavior

**Figure 6:**
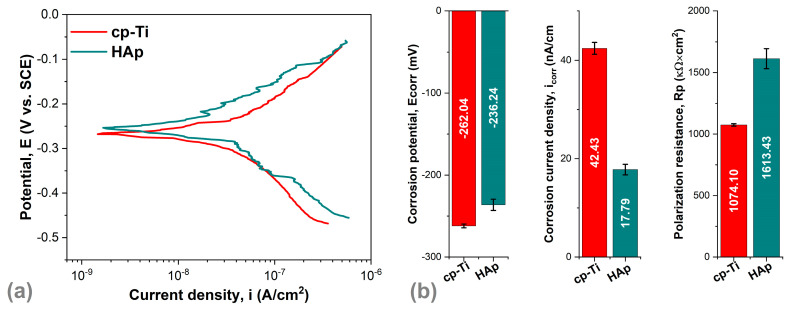
Tafel plots (**a**) and the main electrochemical parameters (**b**) of cp-Ti and the HAp coatings.

Figure 6a shows the Tafel curves of the samples tested in SBF along with the evolution of the main electrochemical parameters obtained from Tafel extrapolation (Figure 6b and Table 2) that characterize the investigated materials in terms of corrosion. 

Although they are the most used today, metallic materials also have disadvantages that place them in the first generation of biomaterials. Among these disadvantages is their susceptibility to localized corrosion, especially pitting corrosion. It is well known that medical implants manufactured from cp-Ti present good corrosion resistance due to the passive film form on the surface. However, the biological solution contains different ions like Na^+^, Cl^−^, and Mg^2+^ that can destroy the passive film. Therefore, in this case, the HAp-based coatings function as a barrier between the biological tissue and the metallic implant, which can prevent the release of corrosion products from the metallic substrate.

An enhanced electrochemical behavior is characterized by an electropositive value of the corrosion potential (E_corr_), a high polarization resistance (Rp), and a low corrosion current density (i_corr_). Considering this, it can be observed that the cp-Ti substrate coated with plate-like crystals registered the best electrochemical behavior, as highlighted by a more electropositive corrosion potential (E_corr_ = −236.24 mV), lower corrosion current density (i_corr_ = 17.79 mA/cm^2^), and higher polarization resistance (Rp = 1613.43 kΩ·cm^2^) than the ones registered by the cp-Ti substrate (E_corr_ = −262.04 mV, i_cor_ = 42.43 mA/cm^2^, Rp = 1074.10 kΩ·cm^2^). The results obtained are in agreement with other studies [24,40], which also showed that the HAp-based coatings with plate-like morphology have enhanced the corrosion resistance of the Ti substrate. 

### 3.7. Biomineralization Assay

**Figure 7:**
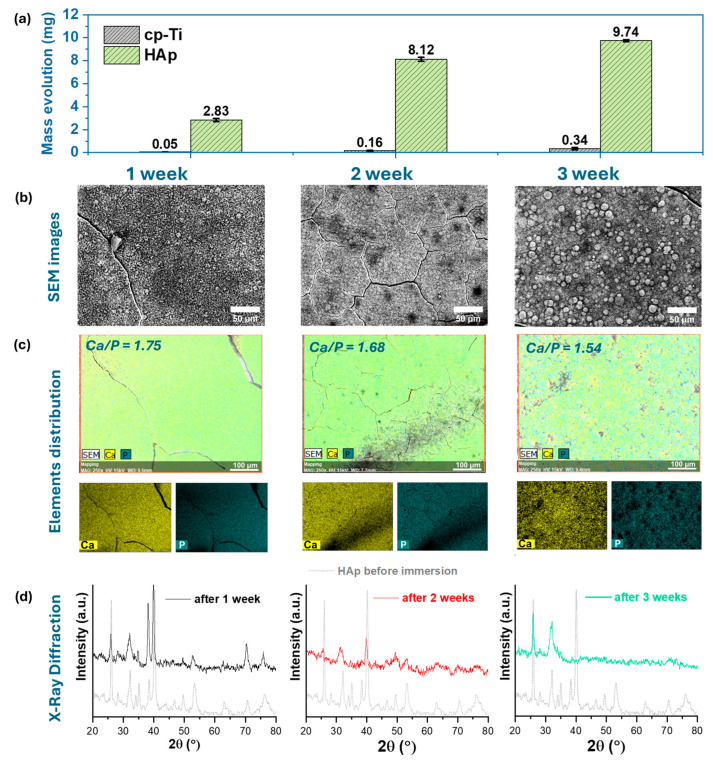
Mass evolution (**a**), SEM images (**b**), EDS results (**c**), and XRD (**d**) after 1, 2, and 3 weeks of immersion in SBF.

The biomineralization tests were performed by immersing the cp-Ti substrate uncoated and coated with HAp in SBF for different periods of time (1, 2, and 3 weeks). For each period, different samples were used. Figure 7 highlights the results obtained after the bioactivity tests, in terms of mass evolution, morphology (3D and 2D-SEM), and chemical composition (EDS). 

In terms of mass evolution of the newly formed apatite (Figure 7a), the obtained results showed that in comparison to the uncoated cp-Ti substrate that gained a mass of only 0.3 mg after 3 weeks of immersion in SBF, the HAp coatings significantly increased their mass recording values of 9.7 mg after the same period. The results obtained for the titanium substrate in terms of biomineralization ability are in good correlation with the literature, indicating that titanium does not favor the precipitation of apatite, while the HAp coatings with plate-like morphology enhanced the biomineralization capacity of cp-Ti in SBF medium [25,40]. 

The results also emerge from the SEM images, presented in Figure 7b, which indicate the formation of a new layer of calcium phosphate regardless of the immersion period. According to the SEM images, the HAp-based coatings present very thin cracks after one week of immersion in SBF, suggesting that the transfer of ions between the HAp coatings and the SBF medium took place rapidly. 

After 2 weeks of immersion in SBF, the newly formed apatite layer is denser and more compact, compared to that after 1 week of immersion. After 3 weeks of immersion, the surface morphology of the newly formed apatite consists of semispherical crystals, which cover the entire surface, highlighting that the proposed coating has a good biomineralization capacity.

The EDS analysis (Figure 7c) revealed that irrespective of the immersion period in SBF, the main elements of HAp, namely Ca and P, were present in the coatings. Regarding the Ca/P ratio, it was noted that this ratio decreases as the period of immersion increases, from a value of 1.75 reached after 1 week of immersion to values of 1.68 and 1.54 after 2 and 3 weeks, respectively. Decrement of the Ca/P ratio during the 3 weeks of immersion in SBF may be attributed to calcium phosphate precipitation, which suggests the formation of Ca-deficient HAp since the obtained values are smaller than those of stoichiometric HAp. 

The XRD analysis (Figure 7d) carried out on the samples immersed for 1, 2, and 3 weeks in SBF showed that the main phase consists of apatite. Nonetheless, in comparison with the HAp coatings before its immersion in SBF (Figure 7c, short dotted line colored in grey), the main peaks associated with HAp (ICDD #09-0432) found at 2θ of 25.88°, 31.77°, 32.20°, and 32.90° are visible, while the ones associated with the titanium substrate (ICDD #44-1294) found at 2θ of 35.09°, 38.42°, and 40.17° begin to diminish after 2 weeks and fully disappeared after three weeks of immersion in SBF medium. Based on this, it can be hypothesized that the HAp coatings along with the newly formed apatite precipitated on their surface led to a thicker and more compact material. 

During immersion in SBF, an ion exchange takes place between the medium and the HAp coatings, through which the calcium ions existing in the coating reach the test environment together with hydrogen. This exchange supports the formation of the OH^−^ group on the surface of the HAp layer. At the same time, the OH^−^ and PO_4_^3−^ groups attract Ca^2+^ ions from the medium to the coatings [103] that subsequently attract the negative phosphate ions (PO_4_^3−^), which in time leads to the formation of crystalline apatite, similar to that found in human hard tissues. 

Thus, the SEM images combined with EDS investigations are in agreement with the XRD analysis, revealing the progressive evolution of the newly formed calcium phosphate phase on the proposed coatings after immersion in SBF.

### 3.8. In Vitro Biocompatibility Assessment

#### 3.8.1. Effect of cp-Ti Substrate HAp Coating on Viability and Proliferation of BMSCs

Calcium and phosphorous ions are incorporated into the microcrystals of HAp found in the bone, leading to matrix deposition on the HAp surface, favoring biointegration [69,104,105]. Aside from designing the chemical structure of the coatings, there is a second possibility to obtain enhanced biointegration, which aids in regulating the stem cell response and osteogenic differentiation. It was demonstrated that cellular behavior is promoted by altering the roughness, morphology, topography, and chemical composition of the coating. In terms of cell–material interaction, the literature [66,106,107] proves that a higher contact area between the cells and the coating enhances cell adhesion, viability, and proliferation.

To evaluate cell viability and proliferation, the viable cells were stained with a fluorescent dye, namely calcein AM, which makes the viable cells green in color, while the dead ones were stained with propidium iodide, which makes the dead cells red in color, indicating the loss of plasma membrane integrity. 

**Figure 8:**
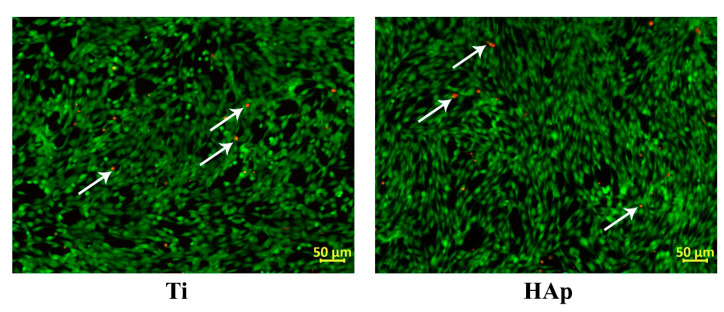
Fluorescence microscopy images—BMSCs cultured for 5 days on Ti and HAp. Viable cells—green; dead cells—red.

**Figure 9:**
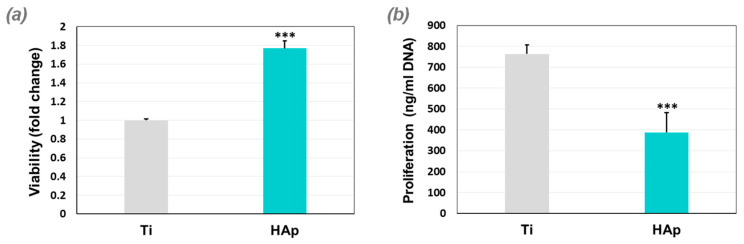
Viability (**a**) and proliferation (**b**) of BMSCs cultured for 5 days on the investigated surfaces (*** *p* < 0.001).

Figure 8 highlights that on both types of surfaces (Ti and HAp coating), most cells are viable (green color), with very few indicated in red, indicating the dead cells. The MTT assays (Figure 9a) confirmed that 5 days after seeding BMSC cells, the highest viability with respect to the DNA amount was obtained by the HAp coatings deposited on the Ti substrate. These results indicate that the HAp coating can support BMSC growth, showing at the same time a negligible reduction in cell viability.

The proliferation tests (Figure 9b) showed that in comparison to the Ti substrate, the HAp-based coatings registered a lower BMSC multiplication after 5 days of cultivation. This result can be explained by the fact that this type of coating can induce cell differentiation towards osteoblasts, but as these cells differentiate, their proliferation capacity decreases. These test results confirmed that a smaller contact angle supports the viability and proliferation of BMSC cells. The higher values reached by the Ti substrate can be explained by its affinity to oxygen ions, which leads to the spontaneous formation of the oxide film that favors cell proliferation [108]. 

#### 3.8.2. BMSC Morphology on cp-Ti Substrate HAp Coating

The in vitro differentiation of BMSCs into osteoblasts is a prolonged process (lasts 21–28 days) that involves three stages: proliferation, differentiation, and maturation. Cell adhesion is a highly specific process that precedes proliferation and differentiation stages. The evaluation of the cell’s adhesion to the cultivation medium after a longer incubation period (24 h) provides relevant information about the cell’s morphology in relation to the surface of the biomaterial with which they interact. In vivo, the integration of the biomaterial at the bone tissue level requires the formation of a stable interface between the extracellular matrix secreted by osteoblasts and the implanted biomaterial surface. This process excludes the formation of any intermediate fibrous tissue between the bone and the surface of the biomaterial [109].

**Figure 10:**
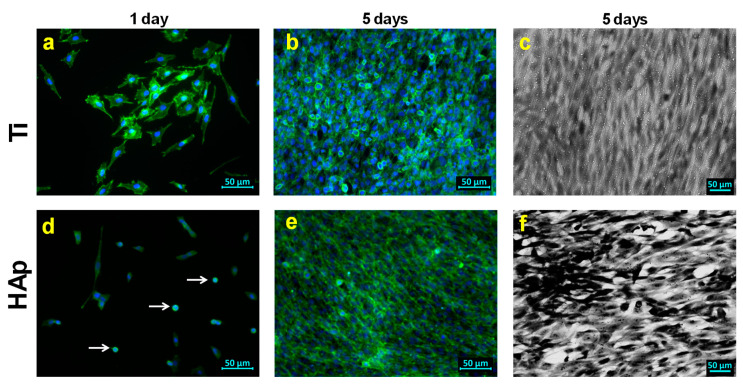
Morphology of BMSCs cultivated on the Ti (**a**–**c**) substrate and HAp (**d**–**f**) coating for 1 day (**a**,**d**) and 5 days (**b**,**e**) in fluorescent images; SEM images after 5 days on Ti (**c**) and HAp (**f**).

Thus, this surface could be considered a stem cell niche that instructs cells to either self-renew or differentiate [110]. The mechanism behind this phenomenon lies in the alteration in the organization of various cytoskeletal components (F-actin, α-tubulin, and γ-tubulin) and with subsequent changes in cell morphology, with a tendency to favor osteogenic differentiation [111]. Furthermore, through these fibers, the nucleus is physically linked to the extracellular matrix and to the surrounding cells. Therefore, by modifying the focal adhesion assembly, intracellular actin polymerization, and cell morphology, the topological features can induce changes in the nuclear matrix through the recruitment of epigenetic modifiers or through the reorganization of chromatin to control stem cell destination [112]. Therefore, it can be noted that at 1 day after cultivation on all tested surfaces, the cells were evenly distributed, and after 5 days they completely covered the surface (Figure 10b,e). 

Furthermore, it was noted that at 1 day after seeding on the HAp coating, the BMSC cells were not perfectly flattened, and some of the cells even kept their round shape (Figure 10d, white arrows) in contrast to the cells seeded on the cp-Ti, which were perfectly flattened. This behavior is due to the differences that appear in the development of focal adhesions in relation to the surface of the substrate [113], especially since it is well known that surface hydrophilicity influences the proliferation and morphology of cells. Thus, as the degree of wetting increases, the cell spreading and size also increase [114]. 

However, after 5 days of seeding, in the case of all analyzed surfaces, the cells were confluent, indicating their multiplication. It is also possible to observe the remarkably close contact between the cells, indicating the presence of contact inhibition, with no differences in terms of colonization degree after 5 days of seeding (Figure 10c,f).

#### 3.8.3. Assessment of Collagen Type I and Fibronectin in BMSCs Grown on cp-Ti Substrate HAp Coating

It is known that the synthesis of matrix proteins also takes place during the multiplication stage. The extracellular matrix plays a significant role by providing structural support and at the same time creating a dynamic microenvironment conducive to cell growth and differentiation [115]. To highlight the main proteins present in the extracellular matrix, namely type I collagen and fibronectin, which are secreted by osteoprogenitor cells/osteoblasts, specific antibodies must be used. The initial antibodies are coupled with secondary antibodies that are fluorescently labeled with Alexa 488 to highlight the proteins in green.

**Figure 11:**
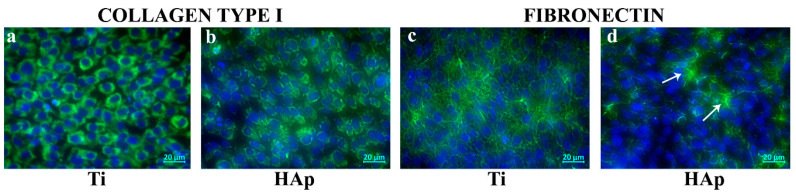
Evidence of collagen type I and fibronectin in BMSCs cultured for 5 days on cp-Ti (**a**,**c**) and the HAp coating (**b**,**d**).

As can be seen in Figure 11a and b, the synthesis of type I collagen by BMSCs is influenced by the cultivation substrate. Thus, it was noted that the BMSCs seeded on cp-Ti and the HAp coating synthesized a very large amount of protein, since approximately 90–95% of the cells were positive for type I collagen, suggesting the ability of these materials to support the osteogenic differentiation process.

Fibronectin is also a protein that functions as a binder between the cells and the collagen from the extracellular matrix. Regarding the synthesis of fibronectin, it was found that unlike type I collagen, it is deposited in certain areas of the HAp coating (Figure 11d, white arrows). This can be explained by the fact that although most cells express type I collagen, it is not yet deposited in the matrix.

#### 3.8.4. Assessment of Alkaline Phosphatase Activity on cp-Ti Substrate HAp Coating

The osteoblast-like function of BMSCs undergoing osteogenic differentiation on the cp-Ti substrate and the HAp coating was evaluated by examining the ALP activity. ALP is known to play a central role in preserving the equilibrium of bone mineralization, which depends on the presence of inorganic phosphate (Pi). This component combines calcium to form HAp crystals and ALP enables this reaction by hydrolyzing inorganic pyrophosphate (PPi) to produce Pi, thus promoting mineralization [116].

**Figure 12:**
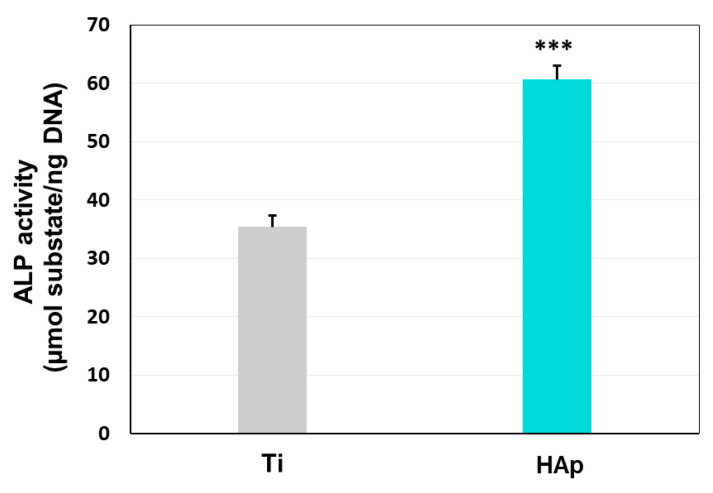
Determination of alkaline phosphatase activity in BMSCs cultured for 5 days on the investigated surfaces (*** *p* < 0.001).

As can be seen in Figure 12, the BMSCs cultured for 5 days on the HAp coatings show the highest enzymatic activity compared to cells cultured on Ti. This result is in accordance with the one obtained regarding cell multiplication. It can be assumed that once the osteoblast differentiation process begins, the cells lose their proliferative capacity, but they begin to present markers specific to bone cells (alkaline phosphatase). High alkaline phosphatase activity is an indication of successful differentiation of BMSCs into osteoprogenitor cells after 5 days of osteoinduction. These results are in accordance with the literature, as the ALP registered better results on surfaces with higher roughness [117].

Nevertheless, these results could be correlated with those obtained in the biomineralization assay that indicated the bioactive character of HAp coatings. Both biomineralization and ALP assays demonstrate the capability of HAp coatings to support the formation of new calcium phosphates on their surface.

## 4. Conclusions

In this work, biomimetic HAp-based coatings with a plate-like morphology were designed by using the electrochemical deposition technique in galvanostatic pulsed mode. The main conclusions are listed below:

- The technique used allows the development of biomimetic HAp coatings made of plate-like crystals, which are wide and thin and grow perpendicular to the Ti substrate;
- Both chemical and phase analysis demonstrated the formation of the HAp phase, with a crystallinity of approx. 26%, a roughness of 228 nm, and a good wetting degree (~17°), indicating a hydrophilic surface that can favor osseointegration;
- The corrosion resistance of titanium was significantly enhanced for the samples coated with HAp in comparison to the uncoated ones, indicating the effectiveness of the coatings to withstand the aggressive attack of the simulated media;
- Unlike bare titanium, the coated samples exhibited promising in vitro biomineralization ability in SBF for up to 3 weeks by stimulating the precipitation of a new layer of Ca-deficient apatite;
- HAp coatings exhibited proper biocompatibility and promoted early in vitro osteogenic differentiation of human BMSCs.

## Figures and Tables

**Table 1:** Parameters involved in the electrochemical deposition of HAp with plate-like morphology summarized from the literature.

Electrolyte (mM)			Potential, E (V)/ Current Density, i (mA/cm^2^)	Deposition Parameters			Pre-/Post- Treatment	Coating Thickness [µm]	Ref.
Ca(NO_3_)_2_ (mM)	NH_4_H_2_PO_4_ (mM)	Others		Time (min.)	Temp. (°C)	pH			
20	12	Yes	E = −1.4 V	120 ÷ 240	60 ÷ 90	4.2	No/No	N/A	[28]
0.61	0.36					6			
0.61	0.36	No	E = −1.4 V	120	85	6	Yes/No	~5	[29]
0.61	0.36	No	E = −1.2 V	180	60	5.6	Yes/No	N/A	[30]
					80			~5	
0.61	0.36	No	E = −1.4 V → 0 V	120	50	5	No/No	1.3	[31]
					75			3.3	
0.61	0.36	No	E = −1.4 V → 0 V	120	75	5	No/No	0.455	[32]
42	25	No	i = 5 ÷ 10 mA/cm^2^	1 ÷ 30	25	4	No/No	N/A	[33]
42	25	Yes	i = 1.25 ÷ 3.61 mA/cm^2^	60	90	N/A	Yes/No	13 ÷ 22	[34]
42	25	No	i = 1 ÷ 20 mA/cm^2^	5 ÷ 40	25	4.11	No/Yes	18.6	[35]
167	100	No	i = 0.375 ÷ 6 mA/cm^2^	60	25	4.6	Yes/Yes	8 ÷ 32	[36]
10	6	No	i = 20 mA/cm^2^	20	30 ÷ 90	N/A	No/No	N/A	[37]
42	25	Yes	i_ON_ = 6 mA/cm^2^; i_OFF_ = 0; t_ON_ = 1; t_OFF_ = 9	15 ÷ 60	65	4 ÷ 8	No/Yes	3 ÷ 14	[38]
42	25	No	i_ON_ = 10 or 20 mA/cm^2^; i_OFF_ = 0 t_ON_ = 1 s; t_OFF_ = 4 s or 8 s	30	65	4.5	Yes/Yes	4 ÷ 31	[39]
42	25	Yes	i_ON_ = 1.5, 5, and 15 mA/cm^2^; i_OFF_ = 0 t_ON_/t_OFF_ = 0.2	25	70	4.3	Yes/No	N/A	[25]
8	5	Yes	i_ON_ = 1.5, 3, and 5 mA/cm^2^; i_OFF_ = 0 t_ON_/t_OFF_ = 0.2			6			
8.4	5	Yes	i_ON_ = 6 mA/cm^2^; i_OFF_ = 0 t_ON_ = 1 s; t_OFF_ = 9 s	30	65	6	Yes/No	6 ÷ 12	[40]
5	3	No	E_ON_ = −1.5 V; E_OFF_ = 0 V t_ON_ = 2 s; t_OFF_ = 2 s	3.33	80	N/A	No/No	1 ÷ 2	[41]
2.5	1.5	No	i_ON_ = −3 mA/cm^2^; i_OFF_ = 0 t_ON_ = 1 s; t_OFF_ = 5 s	30	60	6	No/No	7.5	Current research

**Table 2:** The main corrosion parameters of the cp-Ti substrate and the HAp plate-like coatings.

Sample	E_corr_ (mV)	i_corr_ (nA/cm^2^)	βc (mV)	βa (mV)	Rp (kΩ · cm^2^)
cp-Ti	−262.04 (±2.34)	42.43 (±1.21)	245.07 (±5.08)	183.14 (±4.80)	1074.10 (±10.24)
HAp	−236.24 (±6.85)	17.79 (±1.08)	145.45 (±4.20)	120.86 (±2.83)	1613.43 (±81.25)

## Data Availability

The original contributions presented in this study are included in the article; further inquiries can be directed to the corresponding author/s.
